# Recurrent Anterior Vaginal Wall Hernia after a Radical Cystectomy

**DOI:** 10.1155/2020/8681429

**Published:** 2020-03-20

**Authors:** Omar Felipe Dueñas-Garcia, Kristan Hornsby

**Affiliations:** Obstetrics and Gynecology Department, West Virginia University, Morgantown, West Virginia, USA

## Abstract

True pelvic floor areas are uncommon conditions, but they can occur after extensive pelvic surgery including radical cystectomies or pelvic exenteration. We present the case of a patient with a persistent hernia that failed a native tissue repair and required a prosthetic mesh implant as definitive surgical treatment.

## 1. Introduction

True pelvic floor hernias are rare conditions that are usually seen after extensive pelvic surgery [1]. To our knowledge, this is the second case of this kind of hernia reported in the literature after a radical cystectomy and urethrectomy.

## 2. Case Report

**Figure 1:**
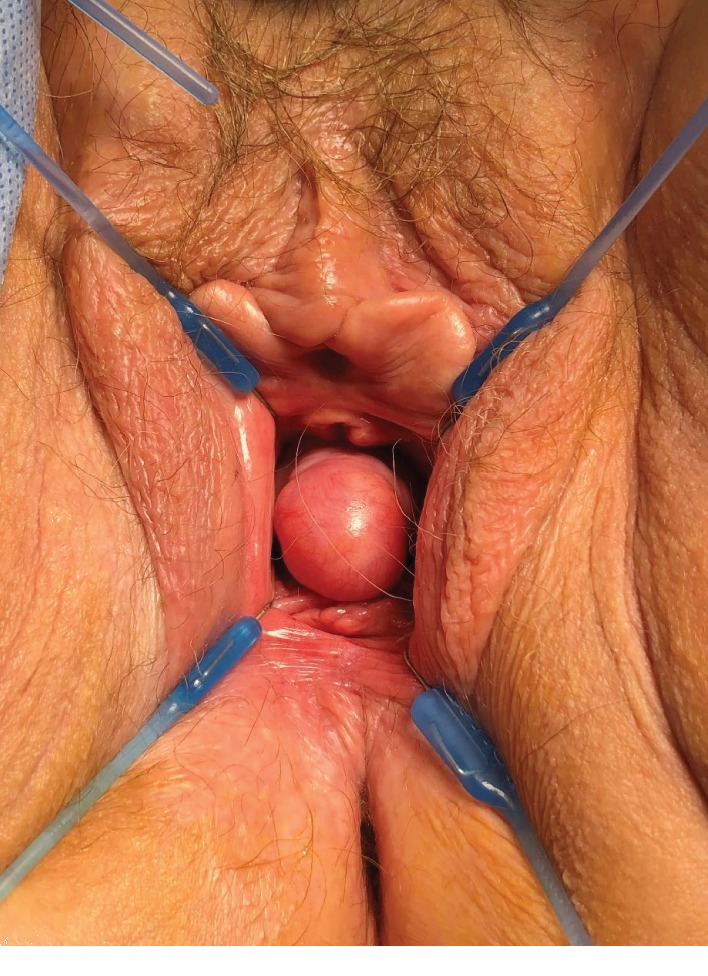
Anterior vaginal wall hernia.

**Figure 2:**
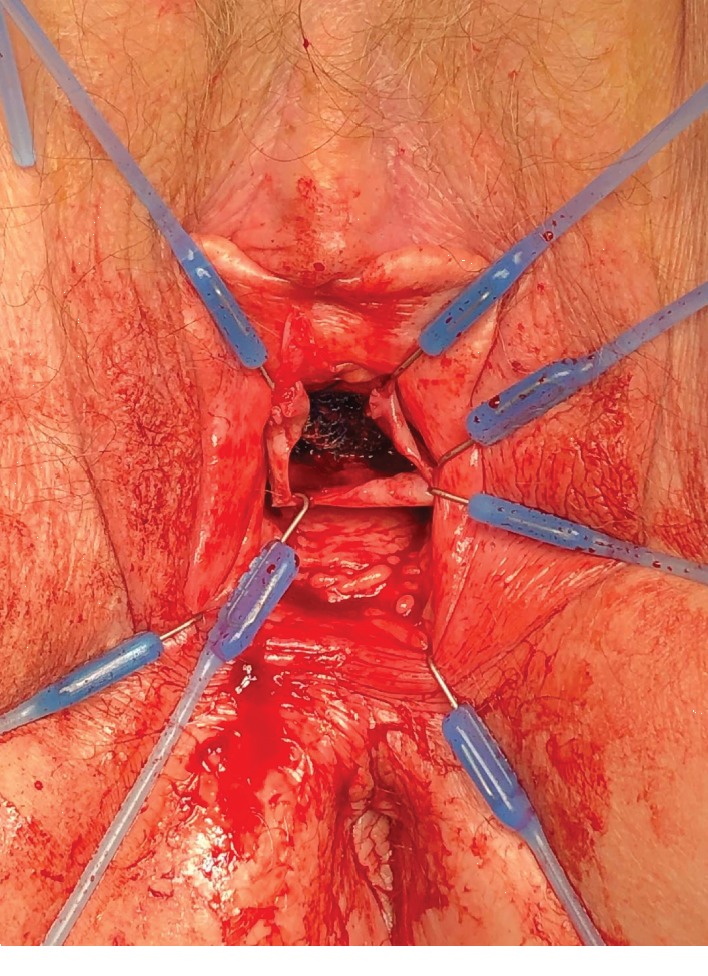
Use of transvaginal mesh to repair the hernia.

**Figure 3:**
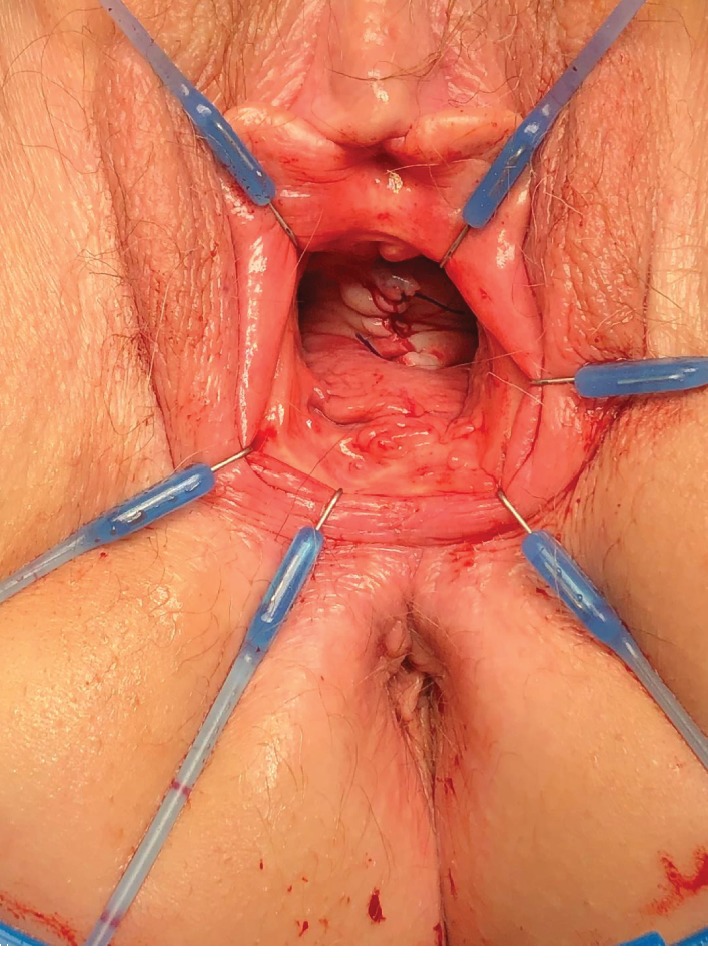
Excellent correction of the anterior wall hernia.

A 74-year-old woman underwent a radical cystectomy, urethrectomy, and ileal conduit at age 70 due to bladder carcinoma. The patient was referred from the urology clinic because of the complaints of having a vaginal bulge sensation. On exam, she was identified with an isolated central anterior vaginal wall defect (true hernia) but with no evident prolapse of the anterior vaginal wall or the vault (Figure 1). The hernia increased its size when performing Valsalva. This problem was causing significant pain, pressure, and discomfort, and she requested permanent solution of her problem. Initially, she underwent an exam under anesthesia with resection of the herniated structure and a native tissue repair. On exam, she had no urethra and access to the peritoneal cavity was performed when resecting the vaginal epithelium and hernia sac. The hernia was sent to pathology with negative findings for malignancy. Unfortunately, the patient had a recurrence of prolapse 4 months after her procedure and she returned to our clinic. The prolapse was more prominent, and a decision was made to perform a transvaginal repair using prosthetic mesh material (Figure 2). We used a type 1 polypropylene mesh from Boston Scientific (Upsylon™ Y-Mesh, Natick, Massachusetts) that was shaped to the size of the defect. The peritoneum was closed using delayed absorbable sutures, and the mesh was anchored using delayed absorbable material to the pelvic floor musculature (Figure 3). After 8 months of follow-up, the patient had excellent support after the completion of the procedure with no signs of recurrence.

## 3. Discussion

Small case series of colorectal exenteration and pelvic exenteration procedures have been reported to cause defects on the pelvic floor. A search in the literature using the PubMed database showed only one previous case of perineal-anterior vaginal wall hernia after a radical cystectomy with urethrectomy [2]. We initially performed a native tissue repair considering her history of malignancy. Unfortunately, the hernia repair only lasted for a couple of months and the patient presented in our clinic again complaining of pain and discomfort. The use of the polypropylene mesh in pelvic reconstructive surgery is extensive with several reports stating that it can improve the anatomical cure and reduce the number of recurrences, especially for the anterior wall compartment [3, 4]. Because previous biopsies and imaging studies showed no evidence of malignancy recurrence, we decided to use a synthetic prosthetic material.

Risk factors for this type of hernia include extensive pelvic surgery, pelvic exenteration, failure to reapproximate the *levator ani* muscles, and tobacco use [5, 6]. In our case, the extensive surgery and debilitated tissue were the possible risk factors for recurrence.

Despite the controversy of using transvaginal mesh, selected use in refractory cases where weakened tissue is encountered remains a viable option to achieve better outcomes.
